# GENERALIZABILITY OF CLINICAL TRIALS OF DELIRIUM INTERVENTIONS

**DOI:** 10.1093/geroni/igz038.1682

**Published:** 2019-11-08

**Authors:** Christina DiBlasio, Mackenzie E Fowler, Roy Martin, Yue Zhang, Richard E Kennedy

**Affiliations:** University of Alabama at Birmingham, Birmingham, Alabama, United States

## Abstract

Delirium, or acute confusional state, affects up to 7 million hospitalized older adults annually, and is associated with increased risk of mortality, institutionalization, and cognitive and functional impairment. There has been a proliferation of both pharmacological and nonpharmacological clinical trials to reduce the incidence and sequelae of delirium. In other neuropsychiatric disorders, exclusion criteria prevent up to 75% of individuals with the condition under study from participating. It is unclear how well these trial samples represent the population of older adults with delirium. We selected all intervention trials registered at ClinicalTrials.gov containing the keyword “delirium” (N=131), regardless of type of intervention. We manually examined study descriptions to restrict analysis to studies with delirium as a primary or secondary outcome (N=92). Of these 92 studies, 76% enrolled only adults, with 45% enrolling only older adults. 38% of studies were restricted to surgical units, 27% to intensive care units, and 7.6% to medical units. Only 1 study examined nursing homes and 4 studies examined palliative care. 50% of studies excluded individuals with pre-existing dementia, 28% excluded individuals with psychiatric disorders, and 30% excluded individuals with neurological disorders. 34% of studies excluded individuals with alcohol or drug abuse. Overall, many intervention studies for delirium are limited to the surgical and ICU populations, and they exclude individuals with common comorbidities associated with an increased risk of delirium. Similar to other neuropsychiatric disorders, these findings raise significant concerns about the generalizability of clinical trials in delirium to the hospitalized older adult population.

